# Polymyositis: A Case Report

**DOI:** 10.7759/cureus.43337

**Published:** 2023-08-11

**Authors:** Mariana Rios-Gomez, Arturo Villanueva-Salinas, Sarahi Arias-Martinez, Juan Andres Pimentel-Esparza, Alejandra Aguirre-Sanchez, Javier Delgado-Villafaña, Martha Elia Perez-Santana, Juan E Montes-Ramirez

**Affiliations:** 1 Internal Medicine, Hospital Regional de Pemex en Salamanca, Salamanca, MEX; 2 Neurology, Hospital Regional de Pemex en Salamanca, Salamanca, MEX; 3 Rheumatology, Hospital Regional de Pemex en Salamanca, Salamanca, MEX; 4 Pathology, Hospital Regional de Pemex en Salamanca, Salamanca, MEX; 5 Neurology, Hospital General de México Dr. Eduardo Liceaga, Mexico City, MEX

**Keywords:** elevated liver transaminases, elevated creatine phosphokinase (cpk), rare muscular disease., inflammatory myopathy, polymyositis

## Abstract

Inflammatory myopathies are a group of diseases whose common pathway is immune-mediated muscle damage, one of which is polymyositis.

The definition of polymyositis is controversial, with proponents advocating a definition based on immunohistochemical and histopathological findings in muscle biopsies, while other proponents advocate a definition based on clinical manifestations and histopathological findings.

Polymyositis is a quite rare disease that is clinically characterized by progressive proximal muscle weakness with a symmetric distribution. Within the diagnostic approach, laboratory studies show elevation of sarcoplasmic enzymes; nerve conduction tests are performed, which may aid in distinguishing myopathic causes of weakness from neuropathic disorders; and muscle biopsy is considered the gold standard to diagnose inflammatory myopathy and to distinguish the subclasses.

We report the case of a 61-year-old male patient who presented generalized symmetrical weakness, predominantly in the upper extremities, and dysphagia, whose laboratory studies, autoantibodies, and muscle biopsy were confirmatory of this entity.

## Introduction

Ernst Leberecht Wagner first described Polymyositis in two publications in 1863 and 1887 [1]. The first diagnostic criteria were established in 1975 by Bohan and Peter (exclude all other myopathies, symmetric proximal muscle weakness, increase in serum muscle enzymes, abnormal electromyographic findings, abnormal muscle biopsy findings, and skin rashes, such as Gottron’s papules) [2] as an inflammatory myopathy whose etiology was not fully understood, it was associated with viral and parasitic infections and non-infectious agents like medications, foods, and biologic agents. It is considered a diagnosis of exclusion; therefore, multiple pathologies must be ruled out before diagnosis. We present a male patient who debuted with symmetrical upper limb weakness and dysphagia and whose laboratory and pathology studies were confirmatory of this rare disease.

## Case presentation

A 61-year-old male patient arrived at the Emergency Room due to a three-week history of progressive generalized weakness and dysphagia of solids. The patient’s weakness predominantly affected the proximal upper limbs symmetrically. The patient had a medical history of type 2 diabetes mellitus treated with 12 units of insulin glargine once daily, diabetic neuropathy treated with gabapentin 300 milligrams once daily, hypertension treated with metoprolol 100 milligrams once daily and nifedipine 30 milligrams twice daily, and end-stage kidney disease on peritoneal dialysis.

Physical examination revealed proximal upper limb weakness, a Medical Research Council Scale (MRC) global strength of 21, diffuse decreased deep tendon reflexes (1+), and normal muscle tone and sensation. The patient was admitted to the hospital for an adequate diagnostic approach.

**Figure 1:**
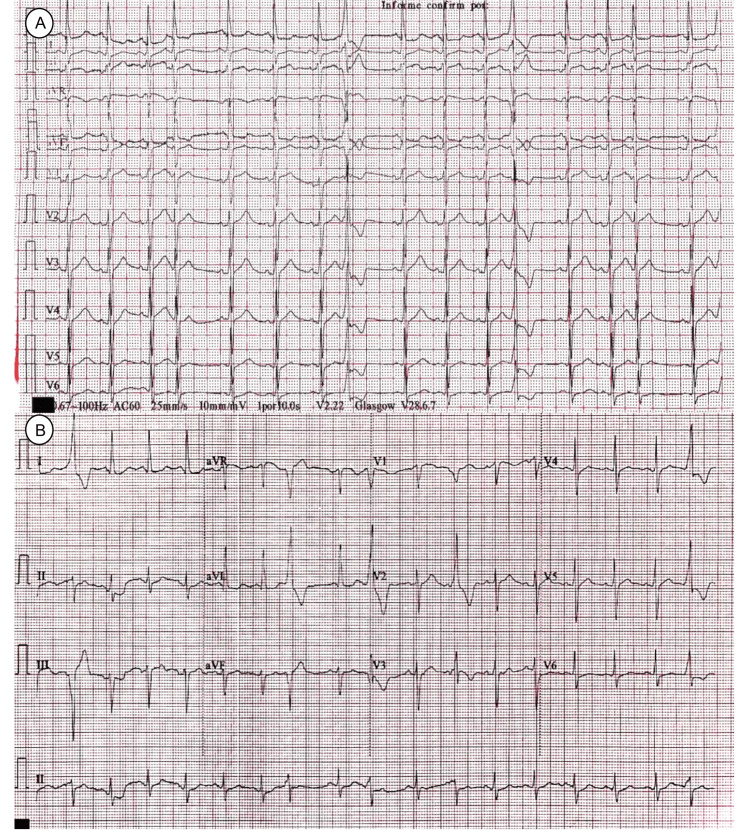
EKG shows isolated premature ventricular contraction (A), EKG evidence T-wave inversion on the anterior leads (B).

The laboratory tests showed leukocytosis of 16.89 10^3/uL (4.6 - 10.4 10^3/uL), but no apparent source of infection could be found (negative cultures). The patient's leukocytosis continued during the hospitalization. To rule out a leukemoid reaction, tumor markers were requested, of which carcinoembryonic antigen 10.2 ng/ml (0-3 ng/ml) and CA-19.9 (142.5 U/ml) (0-35 U/ml) were found to be elevated. A contrasted tomography scan was performed, which didn't show any significant findings. Bone marrow aspirations were taken, and no abnormalities could be found. Other relevant test results were an elevated aspartate transaminase of 208 U/L (10-40 U/L), alanine transaminase of 93 U/L (10-40 U/L), and creatine phosphokinase (CK) of 1521 U/L (26-308 U/L). Due to the cholestatic pattern (R Factor 0.2) found in the liver tests, a liver and bile duct ultrasound was performed; however, no abnormalities were found. Viral panels, including Hepatitis B Virus, Hepatitis C Virus, Human Immunodeficiency Viruses 1 and 2, Cytomegalovirus, and Epstein-Barr Virus, were non-reactive. The laboratory studies during hospitalization can be seen in Tables 1-3 and Figure 1. 

**Table 1:** Laboratories of patients during their hospital stay HDL: High-density lipoprotein; LDL: Low-density lipoprotein; VLDL: Very-low-density lipoprotein.

	Values (Reference values)
Leucocytes (10^3/uL)	16.89 (4.6 to 10.4)
Neutrophils (10^3/uL)	13.4 (2 to 6.9)
Lymphocytes (10^3/uL)	1.5 (0.6 to 3.4)
Monocytes (10^3/uL)	1.5 (0.2 to 0.9)
Eosinophils (10^3/uL)	0.0 (0.04 to 0.54)
Basophils (10^3/uL)	0.3 (0.01 to 0.08)
Erythrocytes (10^6/uL)	3.15 (4.04 to 6.13)
Hemoglobin (gr/dl)	9.3 (12.2 to 18.1)
Hematocrit (%)	31.6 (12.2 to 18.1)
Mean corpuscular volume (fL)	100.3 (80.9 to 97)
Mean corpuscular hemoglobin (pg)	29.6 (27 to 31.2)
Mean corpuscular hemoglobin concentration (gr/dl)	29.6 (31.8 to 35.4)
Red cell distribution width (%)	21.7 (11.7 to 15)
Platelets (10^3/uL)	235 (142 to 424)
Mean platelet volume (fL)	9.5 (9.4 to 12.4)
Glucose (mg/dl)	165 (70 to 105)
Blood urea nitrogen (mg/dl)	52 (10 to 50)
Urea (mg/dl)	111 (17 to 40)
Creatinine (mg/dl)	10.7 (0.5 to 1.2)
Triglycerides (mg/dl)	215 (50 to 200)
Cholesterol (mg/dl)	115 (50 to 200)
HDL cholesterol (mg/dl)	19 (55 to 100)
LDL cholesterol (mg/dl)	35 (151 to 190)
VLDL cholesterol (mg/dl)	43 (8 to 30)
Aspartate aminotransferase (U/L)	208 (10 to 40)
Alanine aminotransferase (U/L)	93 (11 to 41)
Gamma-glutamyltransferase (U/L)	929 (11 to 50)
Lactate dehydrogenase (U/L)	774 (240 to 480)
Alkaline phosphatase (U/L)	1145 (35 to 109)
BIlirrubin (mg/dl)	2.42 (0.1 to 1.18)
Direct bilirrubin (mg/dl)	1.8 (0.05 to 0.3)
Indirect bilirrubin (mg/dl)	0.6 (0 to 0.8)
Albumin (gr/dl)	1.59 (3.5 to 4.94)
Calcium (mg/dl)	8.0 (8.6 to 10.2)
Phosphorus (mg/dl)	5.8 (2.7 to 4.2)
Magnesium (mg/dl)	2.2 (1.58 to 2.55)
Sodium (mmol/L)	132 (134 to 146)
Potassium (mmol/L)	3.6 (3.5 to 5.1)
Chlorine (mmol/L)	90 (98 to 106)
Creatine phosphokinase (U/L)	1521 (26 to 308)
Creatine phosphokinase-MB (U/L)	61 (7 to 25)
Troponin I (ng/ml)	<0.05 (0.0 to 0.4)
Prothrombin time (s)	22.2 (11 to 17)
Partial thromboplastin time (s)	34.7 (24 to 36)
International Normalized Ratio	1.69 (0.8 to 1.2)
Erythrocyte sedimentation rate (mm/h)	65 (0 to 15)
Procalcitonin (ng/ml)	3 (<0.5)
Iron (g/dl)	37 to 158
Vitamin B-12 (pg/dl)	1318 (208 to 963)
Thyroid stimulating hormone (mlU/L)	3.2 (0.5 to 5)
Thyroxine (μg/dL)	6.7 (5 to 12)

**Table 2:** Cerebrospinal fluid (CSF) test results

	Values (Reference values)
CSF Appearance	Clear
CSF Leucocites (cel/uL)	1
CSF Proteins (mg/dl)	48
CSF Glucose (mg/dl)	110
CSF Gram	Non-detected
CSF Culture	Non-detected

**Table 3:** Immunological profile ANA: Anti-nuclear antibodies; SLA: Soluble liver antigen; AMA: Antimitochondrial antibodies; anti-dsDNA Abs: Anti-double-stranded deoxyribonucleic acid antibodies; ACAs: Anti-centromere antibodies; SM: Anti-Smith antibodies; ACPAs: Anti-citrullinated protein antibodies; SRP: Anti-signal recognition particle antibodies.

	Values (Reference values)
Cytomegalovirus IgG	Reactive
Cytomegalovirus IgM	Non-reactive
Epstein-Barr Virus IgG	Non-reactive
Hepatitis B Virus Surface Antigen	Non-reactive
Hepatitis C Virus Antibodies	Non-reactive
Antibodies against HIV-1 and HIV-2	Non-reactive
Lupus anticoagulants (s)	1.08 (<1.2)
ANA	1.11 (<1.5)
SLA	Non detected
Immunoglobulin IgG (mg/dl)	869.91 (552 - 1631)
Immunoglobulin IgM (mg/dl)	124 (40 - 263)
Anti-smooth muscle antibodies	Negative
AMA	0.1 (<1)
anti-dsDNA Abs	10 (<100)
ACAs	<1:40 (<1:40)
Anti–Scl-70 antibodies	Negative
Complement C3 (mg/dl)	74 (81 - 180)
Complement C4 (mg/dl)	16.1 (15 - 57)
Sm antibodies	0.07 (<1)
Anti-Ro/SSA antibodies	0.07 (<1)
Anti-SSB antibodies	0.07 (<1)
ACPAs (U/ml)	<0.5 (0.0 - 5.0)
Anti-Jo-1 antibodies	Negative
Anticardiolipin IgG antibodies (PL-IgG-U/ml)	<2 (<12)
Anticardiolipin IgM antibodies (PL-IgG-U/ml)	<2 (<12)
Anti-beta-2-glycoprotein IgG antibodies	0.31 (<1 )
Anti-beta-2-glycoprotein IgM antibodies	0.37 (<1 )
Antisynthetase-MI-2 antibodies	Non detected
SRP antibodies	Positive (Negative)

**Figure 2:**
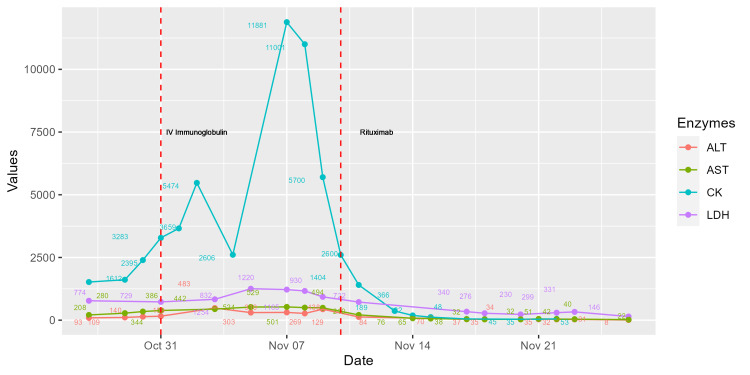
Aspartate aminotransferase (AST), Alanine aminotransferase (ALT), Creatine phosphokinase (CK), and Lactate dehydrogenase (LDH) levels during hospitalization.

**Figure 3:**
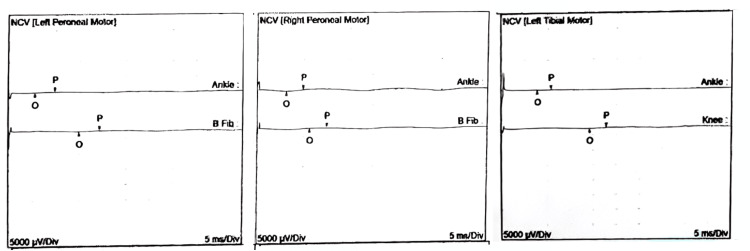
Nerve conduction velocities (NCV): Reduced amplitude in compound muscle action potential (CMAP) and nerve conduction failure consistent with axonal polyneuropathy.

Three days after hospital admission, the patient's upper limb weakness worsened to an MRC of 11, and his dysphagia worsened due to secretion mismanagement. The patient was admitted to the ICU with advanced airway management. A neuromuscular disease was suspected, so a lumbar puncture, nerve conduction velocities (NCV), and electromyography (EMG) were performed at week one of hospitalization. The cerebrospinal fluid (CSF) results were within normal parameters. NCV showed reduced amplitude in compound muscle action potential (CMAP) and nerve conduction failure consistent with axonal polyneuropathy. EMG showed increased activity, positive sharp waves, and fibrillation. During slight muscle contraction, the Motor Unit Action Potential (MUAP) was short duration, polyphasic, and with a small amplitude. These findings should not be present in a demyelinating polyneuropathy (Figure 2). Despite these findings, the ICU decided to use IV Immunoglobulin (24 gr/day) for five days without achieving clinical improvement. The patient developed sinus tachycardia and ventricular extrasystoles. The electrocardiogram (EKG) showed inversion of the T wave on the anterior leads (Figure 3), and his cardiac enzymes showed increased troponin I of 11 ng/ml (0.0 - 0.04 ng/ml). Therefore, management with antiarrhythmic and anti-ischemic drugs was started. Statins were withheld since the patient’s creatine phosphokinase kept rising.

**Figure 4:**
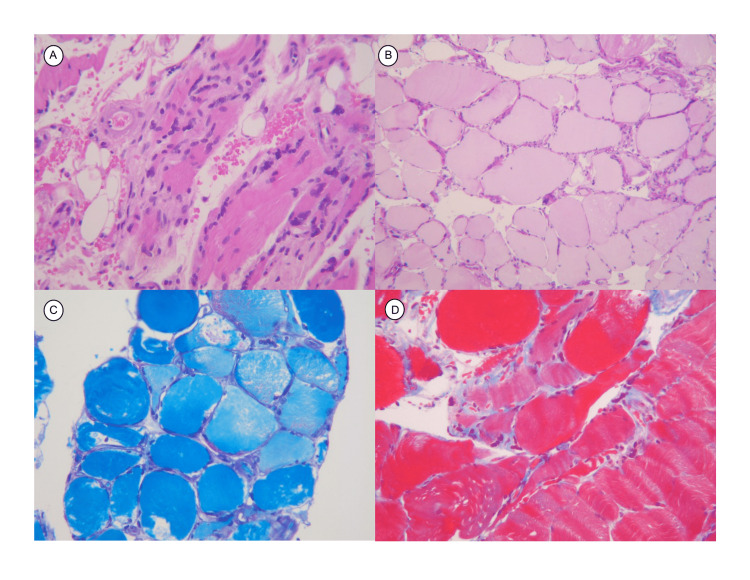
Anterior tibial muscle biopsy. Hematoxylin and eosin stain: chronic inflammatory infiltrate: mature lymphocytes, plasma cells, polymorphonuclear cells, and macrophages. Atrophic fascicles, fatty deposits with fat necrosis and accentuated changes due to regeneration, as well as small and medium-sized vessels with hyperplasia of the media (A). Periodic acid-Schiff (PAS) stain shows the relationship of connective sheaths with fibrosis and disorganization in endomysium, perimysium, and epimysium. Satellite cells are not present (B). Luxol Fast Blue stain shows the absence of neural elements and is positive for the presence of myelin (C). Masson’s trichrome stain shows nuclei peripheral to muscle fibers and fibrosis deposits in the endomysium. It makes the atrophic and wavy muscle fibers more evident (D).

An autoimmune disease was suspected, and antibodies were requested (Table 1). Laboratory tests showed a decrease in Complement C3 and positive anti-signal recognition particle antibodies. These findings suggested an inflammatory myopathy, so treatment with 1 gram of rituximab was initiated, and a biopsy of the anterior tibial muscle was taken. One week after rituximab, the patient showed significant improvement in strength (MRC 18). The muscle biopsy showed endomysial mononuclear and polymorphonuclear infiltration indicative of polymyositis (Figure 4).

Unfortunately, the patient continued on prolonged invasive mechanical ventilation; he developed fever, bilateral lung rattles, and increased oxygen requirements. Ventilator-associated pneumonia due to *Stenotrophomonas Maltophilia* was diagnosed, and treatment with trimethoprim-sulfamethoxazole was started. The patient’s clinical condition worsened, developing disseminated intravascular coagulation, non-variceal upper gastrointestinal tract bleeding, septic shock, multiple organ failure, and eventually death.

## Discussion

Polymyositis (also known as Unverricht-Warner or Wagner-Unverricht syndrome) was first described in the XIX century by Ernst Leberecht Wagner, a German pathologist, and Heinrich Unverricht, a German internist [1]. It was described as a rare idiopathic inflammatory myopathy that was immune-mediated [3].

Polymyositis represents 5% of autoimmune myositis. Furst et al. reported an incidence in the U.S. (United States) of 0.4 to 1.0 per 100,000 person-years [4], and Wilson et al. reported a prevalence of 3.45 per 100,000 inhabitants [5]. Oddis et al. noted that the incidence is higher in black patients [6]. Usually, symptom onset is seen after the second decade of life, with an average age of 50 to 60. This disease is more common in women than in men (2:1). Accurate measurement of incidence and prevalence is challenging due to the diagnostic criteria’s limitations and the lack of inclusion of new myositis-specific autoantibodies [7]. 

Multiple factors have been identified as triggers of inflammatory muscle diseases. A viral etiology (Human T-lymphotropic virus-I, Cytomegalovirus, Influenza virus, Coxsackievirus types A and B, Echoviruses, Parvovirus B19, Hepatitis C virus, Hepatitis B virus, Polio virus, and Human Immunodeficiency Virus) has been proposed by the presence of virus particles and high-titer anti-viral antibodies in patient serum and muscle samples. There have also been case reports where parasitic infections (*Toxoplasma gondii* and *Trypanosoma cruzi*) have been associated with the development of inflammatory myopathies. This association was supported when parasites were found in the muscle lesions, with polymorphonuclear and CD8 T cell infiltration in the muscle biopsy. Furthermore, the patients' myositis symptoms improved after treatment with anti-parasitic drugs. Soft tissue infections by *staphylococci*, *clostridia*, and mycobacteria cause acute muscle inflammation but do not cause chronic, self-sustaining muscle inflammation. In Lyme disease, *Borrelia burgdorferi* has been detected between the muscle fibers, causing inflammation [7,8].

Noninfectious agents related to polymyositis are medications, foods, and biological agents, either as a consequence of a toxic reaction or as an immune-mediated mechanism. Some examples of these agents are D-Penicillamine, fibrates, statins, tiopronin, leuprolide acetate, interleukin-2, interferon-alpha, growth hormone, and fish poisoning (Ciguatera). Noninfectious environmental exposures associated with polymyositis are injections of bovine collagen and chronic graft-versus-host disease. In chronic graft-versus-host disease, the donor's T cells (the graft) view the patient's healthy cells (the host) as foreign and initiate an immune-mediated attack [8].

The pathophysiology of polymyositis is not completely understood, but autoimmune pathogenesis is strongly implicated. Normal muscle fibers do not express MHC (major histocompatibility complex) class I. Nonetheless, activated T cells apparently secrete multiple cytokines such as IFN-γ (interferon gamma), TNF (tumor necrosis factor), and HMGB1 (high mobility group box 1 protein), which induce the expression of MHC I in the sarcolemma of muscle fibers. This sarcolemma is surrounded by CD8+ cytotoxic T cells, forming the CD8-MHC class I complex. These lymphocytes are clonally expanded and release their perforin granules toward the muscle fiber, inducing myofiber damage and necrosis [9].

This MHC class I overexpression leads to endoplasmic reticulum stress through two pathways: unfolded protein response (UPR) and endoplasmic reticulum overload (EOR). UPR turns on endoplasmic reticulum sensors like inositol-requiring enzyme 1 (IRE-1), protein kinase R-like endoplasmic reticulum kinase (PERK) and activating transcription factor 6 (ATF-6) due to glucose-regulated protein 78 (GRP78). These sensors down-regulate the translational machinery and reduce protein accumulation in the endoplasmic reticulum. Also, UPR activation leads to cell death through the CCAAT/enhancer binding protein (C/EBP) and caspase-12, which mediates endoplasmic reticulum-specific apoptosis and cytotoxicity by amyloid-beta. Alternatively, EOR creates a self-sustaining loop by turning on the nuclear factor-κB (NF-κB) pathway, which upregulates the expression of endogenous class I MHC. Thus, more MHC class I is accumulated in the sarcoplasmic reticulum of skeletal muscle fibers, and more NF-κB is activated [10].

The diagnosis of Polymyositis is based on the clinical manifestations, the elevation of sarcoplasmic enzymes (CK, aldolase, lactic dehydrogenase, and transaminases), electromyography, and muscle biopsy [8-11].

The clinical manifestations of polymyositis are progressive symmetrical muscle weakness that develops over weeks or months. The symmetrical weakness is more pronounced in the proximal muscle groups like the neck, shoulders, upper chest, back, arms, and lateral thighs. The lower esophageal sphincter may be affected, causing reflux. When the anal sphincter is affected, fecal incontinence can occur. In more severe cases, patients may develop difficulty swallowing when the throat muscles are affected, which can result in aspiration pneumonia, which can be extremely severe when the diaphragm and breathing muscles are also affected [11]. Fernandez et al. and Schaumburg et al. even reported heart block and cardiac arrhythmias in this disease [12]. 

CK is the most sensitive muscle enzyme and an indicator of the degree of muscle fiber injury. It is elevated in 80%-90% of cases and can increase up to 50 times the upper limit in active disease. However, it does not relate well to the clinical compromise. The values may decrease or normalize without significant clinical improvement or increase without clinical compromise [11].

EMG should always be considered an extension of the clinical examination since it can be misinterpreted. EMG findings include increased insertional activity, polyphasic, short, small MUAP with low amplitude and short duration, positive sharp waves, and high-frequency repetitive discharges with a low level of contraction. These changes are nonspecific but are useful in distinguishing myopathic causes of weakness from neuropathic disorders. It is also used for selecting appropriate muscles for biopsy. 

Most myopathies usually affect the proximal muscles more than the distal muscles. Thus, routine nerve conduction and motor studies are usually normal. However, when the inflammatory myopathy severely affects the recorded muscle reductions and blocks in CMAP may be found. This is due to the fact that the CMAP is a summation of individual muscle action potentials, and these muscles may be affected by inflammatory myopathy [13].

A muscle biopsy is essential since it’s the gold standard for polymyositis diagnosis. The most representative findings are perivascular inflammation, constituted by macrophages and activated CD8+ T cells in muscle fibers, which are concentrated in multiple foci within the endomysium. MHC class I molecule expression is also found in muscle fibers [14].

There are no known specific autoantibodies associated with polymyositis. However, antibodies usually found in this disease are anti-ARS (anti-aminoacyl tRNA synthetase), anti-SS-A (anti-Sjögren’s syndrome-related antigen A), and anti-SRP (anti-signal recognition particle), with a prevalence of 29%, 12%, and 5%, respectively [15].

MRI (magnetic resonance imaging) is the imaging study of choice to assess soft tissues and muscles. MRI can assess inflammation, edema, calcifications, fatty replacement, atrophy, symmetry, and changes in the size of the muscles [16].

Ultrasonography with color Doppler imaging can assess the presence of myopathy. Affected muscles appear hypoechoic due to fatty infiltration and edema, with loss of definition and increased vascularity in the initial phases of the disease [17]. 

Ultrasonography can also be helpful for appropriate muscle biopsy sampling. The limitations of this study are that the abnormal findings in ultrasonography are not specific to inflammatory myopathies and that it’s an operator-dependent study [18]. 

Because polymyositis is a diagnosis of exclusion, other differential diagnoses must be excluded, like neuromuscular diseases and a wide variety of myopathies like dystrophic myopathies, metabolic myopathies, and mitochondrial myopathies, endocrine myopathies, infectious myopathies, drug-induced myopathies. These diseases are discussed in more detail in Table 4 [19-37]. 

**Table 4:** Differential Diagnosis of Polymyositis [20-38]. NSAIDs: nonsteroidal antiinflammatory drugs; SSRIs: selective serotonin reuptake inhibitors; SMA: Spinal muscular atrophy; SMN1: survival motor neuron gene; MG: myasthenia gravis; AChR: acetylcholine receptor; MUSK: muscle-specific kinase; LRP4: lipoproteinrelated protein 4; ACEI: Angiotensin-Converting Enzyme Inhibitors; GAA: acid alpha-1,4-glucosidase; DNA: deoxyribonucleic acid; RNA: ribonucleic acid; CK: creatine kinase; LDH: lactate dehydrogenase; AST: aspartate aminotransferase; FSHD: facio scapulo humeral muscular dystrophy; MM: Miyoshi myopathy; LGMDR2: limb-girdle autosomal recessive muscular dystrophy type 2; DMAT: distal myopathy with anterior tibial onset; DAPC: dystrophin associated protein complex; LGMD: limb-girdle muscular dystrophies; SG: sarcoglycans; DGC: dystrophin-associated glycoprotein complex; AVV: adeno-associated virus; CTM: chronic thyrotoxic myopathy; TPP: thyrotoxicosis with periodic paralysis; ATM: acute thyrotoxic myopathy; IGF-1: insulin-like growth factor 1; OXPHOS: oxidative phosphorylation; PDHc: pyruvate dehydrogenase complex; CSF: cerebroespinal fluid; MRI: magnetic resonance; MRS: proton magnetic resonance spectroscopy; MELAS: mitochondrial encephalomyopathy, lactic acidosis, and stroke-like episodes syndrome; tRNA: transfer ribonucleic acid; MERRF: myoclonic epilepsy with ragged red fibers; HMG-CoA: 3-hydroxy-3-methylglutaryl coenzyme A; AZT: zidovudine; mtDNA: mitochondrial DNA; AIDS: acquired immunodeficiency syndrome.

	Epidemiology	Physiopathology	Clinical features.	Diagnosis	Treatment	Prognosis
Neuromuscular diseases						
Motoneuron Diseases. Amyotrophic lateral sclerosis (ALS)	*Incidence:2 per 100,000 individuals worldwide. *Mean age of onset: 55 - 60 years. *More commonly affects men than women.	* ≥ 30 genes confer major risk of ALS. *C9orf72, TARDBP, SOD1 and FUS are the genes responsible for 70% of cases. *Aggregation and accumulation of ubiquitylated proteinaceous inclusions in motor neurons. *Spinal, bulbar and cortical motoneurons become dysfunctional and degenerate.	*Cognitive impairment. *Behavioral impairment. *Dysphagia. *Dysarthria. *Respiratory insufficiency. *Muscle cramps. *Spasticity. *Muscle weakness. *Muscle atrophy.	*El Escorial criteria. *Airlie House criteria	*Disease-modifying therapies: -Riluzole. -Edavarone. *Symptomatic: -Spasticity: baclofen and tizanidine. -Sialorrhoe: anticholinergic drugs,amitriptyline, glycopyrrolate, botulinum toxin A or B. -Pain: gabapentin, pregabalin, tricyclic antidepressants, NSAIDs, opioids, intra-joint injections of lidocaine or steroids. -Muscle cramps: quinine sulfate, levetiracetam, mexiletine. -Dysphagia: dietary changes, exercises, gastrostomy, parenteral nutrition. -Dysarthria:speech therapy,brain-computer interfaces -Deep venous thrombosis: compression stockings and anticoagulation therapies. -Mood alterations: SSRIs, tricyclic antidepressants, dextromethorphan, quinidine. -Cognitive impairment: SSRIs and antipsychotics may help. -Respiratory insufficiency: noninvasive ventilation.	*Average survival from symptom onset is 3 years. *King’s system.*Milano - Torino system.
Motoneuron Diseases. Spinal muscular atrophy	*Most common form of motoneuron disease in children and young adults. *Incidence:1 in 11,000 live births.	*90% caused by an homozygous deletion or mutation in the 5q13 SMN1 gene.	*SMA type 0: decreased fetal movements, neonates with severe weakness and hypotonia, areflexia, facial diplegia, atrial septal defects, joint contractures and respiratory failure. *SMA type 1: hypotonia, poor head control, reduced or absent tendon reflexes before 6 months of age, tongue and pharyngeal muscles weakness, aspiration and respiratory failure. *SMA type 2: progressive proximal leg weakness greater than weakness in the arms, never able to walk independently, orthopedic complications, hypotonia, areflexiae, restrictive lung disease. *SMA type 3: progressive proximal weakness of the legs more than the arms, may need a wheelchair. *SMA type 4: similar to SMA type 3, mildest form of the disease	*Molecular genetic testing: detection of homozygous deletions of exons 7 of the SMN1 gene.	*Pulmonary: early implementation of noninvasive ventilatory support, tracheostomy, permanent ventilatory support. *Gastrointestinal: dietary changes, early gastrostomy, Nissen fundoplication if gastrointestinal reflux is present. *Orthopedic: physiotherapy. *Disease-modifying therapy: nusinersen, onasemnogene abeparvovec and risdiplam.	*SMA type 0: 6 months life expectancy. *SMA type 1: 2 years life expectancy. *SMA type 2: > 2 years of life expectancy. *SMA type 3: life expectancy not affected. *SMA type 4: life expectancy not affected.
Myasthenia gravis	*Worldwide prevalence of 40-180 per million people. *Annual incidence of 4-12 per million people. *2 peaks: young adults (30 years) and older than 50 years.	*B-cell mediated. *Autoantibodies against AChR, MUSK and LRP4.	*Weakness in the extraocular, bulbar, limb, and axial muscles. *Ptosis. *Diplopia. *Weakness of external eye muscles is nearly always asymmetrical. *Limb weakness is symmetrical and more proximal than distal.	*Ice pack test. *Laboratory findings: autoantibodies against AChR, MUSK and LRP4 (seronegative 6%). *Nerve conduction testing with repetitive nerve stimulation: progressive reduction in the amplitude of the compound muscle action potential. *Single-fiber electromyography: increased jitter.	*Symptomatic: pyridostigmine, neostigmine, ambenonium chloride. *Immunosuppressive treatment: prednisone, prednisolone, azathioprine, mycophenolate mofetil, rituximab, ciclosporin, methotrexate. *Myasthenia gravis crisis: intravenous immunoglobulin and plasma exchange are specific immunosuppressive.	*MG Activities of Daily Living score. *MG Manual Muscle Testing. *MG Composite Scale. *Quantitative MG scoring system.
Metabolic myopathies						
McArdle’s disease	*Prevalence of 1:167,000 in the Spanish population. *Equally in both sexes. *Early or late childhood, sometimes adulthood.	*Autosomal recessive mutations in both alleles of the PYGM gene, which encodes myophosphorylase, the skeletal muscle isoform of glycogen phosphorylase. These mutations result in a lack of functional mature protein. *Muscle glycogenolysis blocked.	*Exercise intolerance (static or isometric muscle contractions and dynamic exercises). *Improved exercise tolerance after ingesting carbohydrates. *Acute exercise can trigger crises with excessive fatigue, contractures, rhabdomyolysis and myoglobinuria. *”Second wind” phenomenon.	*Laboratory findings: Increased levels of serum CK at rest. *Molecular genetic testing: mutations in both PYGM alleles. *Negative histochemical reaction for myophosphorylas. *No myophosphorylase activity.	*ACEI inhibitors, dantrolene sodium. *Diet rich in complex carbohydrates.	*Not life threatening. *Risk of acute kidney injury due to rhabdomyolysis.
Pompe's disease	*Usually presents in patients within the first months of life. *1 in 40,000 in African-American. *1 in 50,000 in Chinese. *1 in 40,000 in Dutch. *1 in 146, 000 in Australian.	*Autosomal-recessive with allelic heterogeneity in the gene 17q25.2-q25 encoding lysosomal GAA. *GAA deficiency leads to accumulation of glycogen within the lysosomes and affects lysosomal-mediated degradation of glycogenesis.	*Infantile-onset form: cardiomyopathy, hypotonia, cardiomegaly, respiratory distress, muscle weakness, feeding difficulties, failure to thrive, hepatomegaly. *Late-onset form: do not develop cardiomyopathy, may present at any age, involvement of the diaphragm, sleep-disordered breathing, respiratory failure.	*Laboratory findings: increased levels of serum CK, LDH and AST. *Deficiency of GAA activity. *DNA analysis. *Electromyography: myopathic discharges, myotonic and repetitive discharges. *Forced vital capacity is reduced substantially.	Enzyme replacement therapy with alglucosidase alfa.	*Without treatment, rarely survive beyond 1 year of age.
Dystrophic myopathies						
Facio scapulo humeral muscular dystrophy	*Prevalence: between 1:15,000 and 1:20,000. *Third most common form of muscular dystrophy. *Late teens to early 20s.	FSHD 1: *95% have a critical number loss of macrosatellite repeats (D4Z4) in the subtelomeric region of chromosome 4q35. *Contraction in the number of D4Z4 repeats results in chromatin relaxation and transcriptional de-repression of DUX4 (double homeobox gene). FSHD 2: *5% don’t have contraction in the D4Z4 repeats. *Inappropriate expression of the DUX4 gene. *Mutations in SMCHD1 (gene on chromosome 18) are involved in chromatin regulation. *Caspase-3-mediated apoptosis, negatively affect myogenesis. *p53-mediated toxicity.	*Weakness starts in the face (orbibularis oculi and orbicularis oris are the most affected muscles) and advances asymmetrically to the scapular muscles until it affects trunk and lower extremities muscles. *10% develops restrictive lung disease. *Hearing loss. *Retinal vascular disease.	*Genetic testing: FSHD1 and FSHD2. *Electromyography: shows myopathic changes, findings are nonspecific.	*Antioxidants like vitamin E, vitamin C, zinc gluconate, and selenomethionine. *Physical rehabilitation.	*Life expectancy is generally unaffected.
Dysferlinopathies	*Late teens to early 20s. *Late onset at 70s has been reported.	*Mutations in the DYSF gene in chromosome 2p13.2 which encode dysferlin protein. *Dysferlin repairs defects in skeletal membrane by regulating vesicle fusion with the sarcolemma and maintains integrity of the muscle membrane.	*MM: -Atrophy and weakness of the posterior calf muscles. *LGMDR2: -Weakness of the proximal thigh muscles. *DMAT: -Weakness of the anterior tibial muscle, subsequently affects proximal muscles of lower and upper extremities. *Isolated hyperCKemia: -Considered as the prelude of other forms of dysferlinopathies.	*Electromyography: fibrillation potentials, positive sharp waves, normal motor and sensory nerve conduction tests. *Muscle biopsy: variability in fiber size, increased endomysial connective tissue, mononuclear inflammatory cell infiltrate in the endomysium and perivascularly, scattered necrotic and regenerating fibers. *Western blot: reduction or absence of dysferlin. *Laboratory findings: high levels of CK.	*Physical and occupational therapy.	*Poor, slowly progressive disease. *Unable to walk within approximately 10 years of symptoms onset.
Becker’s dystrophy	*1 in 5,000 to 1 in 6,000 live male births. *Prevalence: 10 cases per 100,000 males. *Very rare in females (<1 per million). *Age of onset varies widely from 5 to 60 years of age.	*Inherited as X-linked recessive traits. *Mutations in DMD gene in the Xp21.2 chromosome (which encodes dystrophin protein). *Dystrophin deficiency results in the disassembly of the DAPC which leads to sarcolemma weakening, consequently, there is muscle damage during its contraction.	*Clinical presentation and onset of symptoms varies widely. Growth delay. *Progressive muscle weakness affecting proximal earlier than distal limb muscles. *Cognitive impairment. *Fractures. *Cardiomyopathies. *Atrophy and pseudohypertrophy of calf muscles.	*Laboratory findings: high levels of CK. *Electromyography. *Muscle biopsy: shows necrosis, regeneration, fatty replacement, and endomysial fibrosis. *Genetic testing.	*Multidisciplinary care. *Corticosteroids (prednisolone or deflazacort).	*Life expectancy is 40 to 50 years. *Death due to dilated cardiomyopathy.
Myotonic dystrophy type 2	*Prevalence varies globally. *Age of onset varies from 20 to 70 years of age.	*Expansion of a CCTG tetramer in CNBP gene on chromosome 3q21.3. *Expanded DNA is transcribed into expanded RNA, interfering with RNA biogenesis and gene expression. *CNBP gene is expressed in multiple tissues resulting in a multisystem disease.	*Weakness of neck flexion, truncal muscles, proximal leg and arm muscles. *Myotonia in proximal muscles. *Myalgia which worsens with cold weather, pressure and exercise. *Respiratory impairment. *Cardiac conduction abnormalities. *Cognitive deficits. *Gastroesophageal reflux and constipation. *Cataracts. *Insulin resistance and dyslipidemia.	*Genetic testing. *Electromyography: shows myopathy and electrical myotonia. *Muscle biopsy: shows myopathy with bountiful pyknotic clumps and central nuclei.	*Multidisciplinary care	*Life expectancy is 80 years.
Sarcoglycanopathies	*Prevalence around 1/178,000 habitants *Onset in the first decade of life.	*Four subtypes of autosomal recessive LGMD: LGMDR3, LGMDR4, LGMDR5 and LGMDR6 caused by mutations in the SGCA, SGCB, SGCG and SGCD genes which encodes α-SG, ß-SG, λ-SG and δ-SG. *These sarcoglycan proteins are members of the DGC which provides mechanical support to the sarcolemma during muscle contraction.	*Calf hypertrophy. *Progressive muscle weakness. *Cardiac and respiratory impairment with disease progression.	*Laboratory findings: high levels of CK. *Muscle biopsy: shows abnormal staining for all four sarcoglycans.	*Multidisciplinary care. *Gene therapy using AAV.	*Leads to significant disability during childhood.
Endocrine myopathies						
Thyrotoxic myopathy	*CTM and TPP are the most common. *TPP is more prevalent in Asians, Latinos and Caucasians, age of onset from 20 to 40 years and male sex is the most affected.	*Is a complication of hyperthyroidism. *Excess of thyroid hormone reduce CK activity and the content of creatinine and phosphate in the skeletal muscle. *Thyroid hormone impacts the mitochondria of myocytes, causing swelling, degeneration and damage to cell metabolism, inducing weakness and muscle atrophy. *In TPP, defects in Kir channels on skeletal muscle cell membranes are found.	*CTM: -Symmetrical limb weakness. -Proximal muscle involvement (scapular girdle muscle is more affected) -Bulbar paralysis is rare. *TPP: -Paroxysmal muscle weakness affecting distal limb muscles. -Dyspnoea due to intercostal and diaphragmatic paralysis. -TPP attacks are frequent at night (hypokalaemia, hypophosphataemia and mild hypomagnesaemia). *ATM: -Rapidly progressive severe muscle weakness. -Bulbar paralysis	*CTM: -Serum potassium, CK, and myoglobin levels are normal. *TPP: -Kir2.6 mutation. -Hypokalaemia, hypophosphataemia and hypomagnesaemia. *Laboratory findings: increased or decreased thyroid-stimulating hormone, and decreased thyroxine levels.	*CTM: -Anti-thyroid drugs. *TPP: -Potassium supplementation. -b-blockers *ATM: -Must be treated as an hyperthyroidism crisis.	*If untreated can be an extremely debilitating disorder. In rare cases lead to death.
Cushing’s syndrome	*Prevalence: 42-83% in patients with Cushing’s syndrome. *Higher prevalence in ectopic Cushing’s syndrome and in male patients.	*Muscle weakness due to glucocorticoid-induced inhibition of protein synthesis. *Glucocorticoids repress IGF-1, inhibits aminoacids uptake, down regulates myogenin, stimulates myostatin and proteolysis.	*Muscle pain and weakness first affecting the lower extremities and then the upper extremities. *Severely affects the proximal part of lower limbs. *Acute bilateral carpal tunnel syndrome. *Physical features of hypercortisolism.	*Laboratory findings: normal levels of CK. *Electromyography: decrease in muscle fiber conduction velocity. *Muscle biopsy: atrophy of type 2B muscle fibers.	*Treatment directed to the etiology of the Cushing’s syndrome. *Adrenal-directed agents: ketoconazol. *Pituitary-directed drugs: pasireotide. *Glucocorticoid receptor-directed drugs: mifeprostone. *Pituitary or adrenal tumor removal	*Take months to years to resolve
Mitochondrial myopathies						
Subacute necrotising encephalopathy or Leigh syndrome	*Debuts in the first years of life, although it has also been reported in late childhood and adulthood.	*Genetic impairment of the mitochondrial pathways of energy generation. *Mutations in more than 75 genes have been described, these genes encode structural components of the OXPHOS pathway and the PDHc which leads to mitochondrial dysfunction.	*Muscular hypotonia, spasticity or dystonia,. *Seizures. *Ataxia. *Dysphagia. *Ptosis, nystagmus or slow saccades. *Respiratory impairment. *Psychomotor retardation. *Feeding difficulties. *Sensorineural deafness. *Hypertrichosis in extremities and forehead.	*Laboratory findings: lactic acidosis, increase of the lactate/pyruvate ratio in blood, urine and CSF, hyperalaninemia, urinary organic acids, carnitine panel. *Cerebral MRI: bilateral, symmetrical hyperintensities in T2-weighted in the basal ganglia and brain stem. *MRS: lactate peak in the brain parenchyma or the CSF. *Fibroblast culture. *Muscle biopsy. *Mitochondrial DNA screening.	*Supportive care. *Coenzyme Q10. *Thiamine. *Biotine. *Riboflavin *L-Carnitine. *Vitamins C and E. *High-fat diet. *Creatine monohydrate.	*Typically results in death within two to three years, due to respiratory failure.
Mitochondrial encephalomyopathy, lactic acidosis, and stroke-like episodes syndrome	*Prevalence in Japan: 0.2:100,000.	*m.3243ANG mutation in the MT-TL1 gene which encodes the mitochondrial tRNA, resulting in impaired mitochondrial translation and protein synthesis leading to mitochondrial dysfunction. *Due to energy deficiency, mitochondria proliferates in the smooth muscle leading to endothelial dysfunction, angiopathy and impaired blood perfusion in the microvasculature.	*Stroke-like episodes (hemiparesis, hemianopia, cortical blindness). *Dementia. *Epilepsy. *Lactic acidemia. *Recurrent headaches. *Hearing impairment. *Diabetes. *Short stature. *Exercise intolerance. *Muscle weakness. *Motor developmental delay.	*Hirano’s diagnostic criteria for MELAS. *Electromyography: axonal or mixed axonal and demyelination neuropathy. *Muscle biopsy: ragged red fibers in skeletal muscle. *Genetic testing.	*Multidisciplinary care. *L-arginine. *Citrulline. *Coenzyme Q10. *Creatine monohydrate. *L-Carnitine.	*Median survival rate from onset is 8.65 years. *Acute stroke-like episode and/or status epilepticus are the predominant causes of death
Myoclonic epilepsy with ragged red fibers	*Prevalence in Northeast England:0.7:100,000.	*A to G mutation at nucleotide 8344 in the mitochondrial MT-TK gene encoding tRNA (Lys). *Transmitted by maternal inheritance.	*Myoclonus. *Generalized epilepsy. *Ataxia. *Myopathy. *Exercise intolerance. *Dementia. *Ptosis. *Sensorineural hearing loss. *Short stature. *Optic atrophy. *Peripheral neuropathy. *Cardiomyopathy. *Pigmentary retinopathy. *Pyramidal signs. *Ophthalmoparesis *Lipomas	*Laboratory findings: lactic acidosis in blood and CSF, also protein levels may be increased in CSF. *Electromyography: often normal but may show myopathic features. *Muscle biopsy: ragged red fibers in the modified Gomori trichrome stain and hyperactive fibers in the succinate dehydrogenase stain. *Genetic testing.	*Multidisciplinary care. *Levetiracetam. *Clonazepam. *Ubiquinol. *Carnitine. *Alpha lipoic acid. *Vitamin E. *Vitamin B complex. *Creatine	*Exact survival rate is unknown, but patients requires routine evaluations every 6 to 12 months.
Drug-induced myopathies						
Corticosteroid myopathy	*60% of patients using glucocorticoids may develop myopathy. *Dose dependent. *More frequent with dexamethasone, betamethasone and triamcinolone.	*Direct catabolic effect: glucocorticoids decrease protein synthesis and increase protein catabolism. Inhibit IGF-I and leucine in the phosphorylation of eIF4E-binding protein 4E-BP1 and ribosomal protein S6-kinase 1. *Downregulate myogenin. *Activate cathepsins and calpains. *Mitochondrial dysfunction.	*Acute form. -Often in the intensive care unit. -Rapidly progressive proximal and distal muscle weakness. -Respiratory muscles can also be affected. -Recovery can take months. *Chronic form. -Progressive and painless weakness that mostly affects proximal muscles. -Recovery in weeks. -Features of Cushing’s syndrome.	*Laboratory findings: muscle enzymes levels are normal or slightly high, aldolase and aminotransferases levels are normal. In the acute form, CK can rise 50%. *Electromyography: in the chronic form, may show myopathic features. *Muscle biopsy: nonspecific atrophy of type IIb muscle fibers.	*Minimize dose and duration of glucocorticoid exposure or discontinue if possible. *Replace fluorinated glucocorticoids with non fluorinated glucocorticoids.	*3 to 4 weeks after discontinuation of the glucocorticoid, muscle strength improves.
Statin myopathy	*1 of 10,000 treated persons per year. *Dose dependent. *Recurrence risk. *More frequent with lovastatin, simvastatin and atorvastatin which are metabolized by cytochrome P450 3A4.	*Autoantibodies against HMG-CoA reductase. *DRB1*11:01 is associated in the development of these autoantibodies. *Statin binds to HMG-CoA reductase changing protein conformation which leads to the generation of cryptic epitopes.	*Onset of muscle symptoms may occur at any time during treatment. *Progressive symmetrical proximal muscles weakness. *Statin-associated muscle symptoms clinical index (SAMS-CI).	*Laboratory findings: CPK levels ≥10 times the upper limit of normal, autoantibodies against HMG-CoA reductase. *MRI: muscle edema. Electromyography: myopathic features. *Muscle biopsy: muscle-cell necrosis and regeneration, cellular infiltrates in endomysial and perivascular regions.	*Discontinuation of statins. *Prednisone. *Methotrexate. *Azathioprine. *Mycophenolate mofetil. *Intravenous immune globulin. *Rituximab.	*After discontinuation of the statin, muscle strength improves.
Zidovudine myopathy	*17% of patients using zidovudine may develop myopathy. *Especially seen after a lifetime dose of AZT > 200 g.	*mtDNA depletion due to inhibition of DNA polymerase. *Oxidative stress. *Direct inhibition of mitochondrial bioenergetic machinery. *Mitochondrial depletion of L -carnitine. *Myofiber apoptosis.	*Progressive generalized muscle pain, and weakness. *Muscle atrophy.	*Laboratory findings: increased CK levels. *Muscle biopsy: ragged red fibers, cytochrome oxidase negative fibers, structurally abnormal mitochondria.	*Discontinuation of zidovudine. *Carnitine.	*After discontinuation of zidovudine, myopathy rapidly improves.
Infectious myopathies						
HIV myopathy	*25% of AIDS patients.	*Dysregulation of T cells leads to muscle fiber inflammation and insufficient muscle repair. *Sometimes associated with immune reconstitution syndrome.	*Subacute onset of proximal symmetric muscle weakness. *Myalgias.	*Laboratory findings: increased CPK levels. *Muscle biopsy: red-rimmed vacuoles, endomysial T-cell infiltrates, cytoplasm inclusions, atrophic fibers, amyloid deposits	*Steroids. *Methotrexate. *Azathioprine. *Intravenous immune globulin. *Cyclophosphamide. *Cyclosporine.	Good prognosis

High-dose glucocorticoids are the initial treatment for polymyositis. Oral prednisone 1 mg/kg is recommended, and in severe and rapidly worsening disease, intravenous methylprednisolone 1 g/dose once daily for 3-5 days may be used. Disease-modifying antirheumatic drugs (DMARDs) must be used to reduce the side effects of steroids and enhance their immunosuppressive effect. Some DMARDs used are mycophenolate mofetil, which inactivates inosine monophosphate dehydrogenase, an important enzyme in purine synthesis, which in consequence, inhibits T and B-cell proliferation (2-3 g per day divided into two doses), methotrexate, which inhibits folic acid and purine metabolism and adenosine signaling (10-25 mg per week orally or subcutaneously), and azathioprine, a purine analog that blocks T and B-cell proliferation (2-3 mg/kg per day) [38].

In patients with a rapidly worsening disease, severe life-threatening weakness, dysphagia with a high risk for aspiration, or resistant disease, intravenous immunoglobulin G (IVIG) is recommended (2 g/kg administered in divided doses over 2 to 5 days every four weeks for 3-6 months). When glucocorticoids and intravenous immunoglobulin are not effective, treatment escalation with rituximab, a monoclonal antibody against CD20, should be considered (1 g once every two weeks for two doses) [39].

## Conclusions

Polymyositis is a rare disease whose etiology is not fully understood and requires a meticulous diagnostic approach. There have been few cases reported to date, so full documentation and publication are of paramount importance. This will help us improve the detection of this rare disease, achieve a better understanding of the factors associated with it, and improve its characterization. Also, we want to highlight that this pathology may have significant morbidity and mortality. Thus, the adequate interpretation of NCV and EMG is of paramount importance. Incorrect interpretation can lead to mismanagement, delayed treatment, disability, a high risk of complications related to management, and death.
